# Orthodontic Forces Induce the Cytoprotective Enzyme Heme Oxygenase-1 in Rats

**DOI:** 10.3389/fphys.2016.00283

**Published:** 2016-07-19

**Authors:** Christiaan M. Suttorp, Rui Xie, Ditte M. S. Lundvig, Anne Marie Kuijpers-Jagtman, Jasper Tom Uijttenboogaart, René Van Rheden, Jaap C. Maltha, Frank A. D. T. G. Wagener

**Affiliations:** Department of Orthodontics and Craniofacial Biology, Radboud university medical centre, Radboud Institute for Molecular Life SciencesNijmegen, Netherlands

**Keywords:** orthodontic tooth movement, alveolar bone remodeling, cytoprotective enzymes, hyalinization, root resorption

## Abstract

Orthodontic forces disturb the microenvironment of the periodontal ligament (PDL), and induce craniofacial bone remodeling which is necessary for tooth movement. Unfortunately, orthodontic tooth movement is often hampered by ischemic injury and cell death within the PDL (hyalinization) and root resorption. Large inter-individual differences in hyalinization and root resorption have been observed, and may be explained by differential protection against hyalinization. Heme oxygenase-1 (HO-1) forms an important protective mechanism by breaking down heme into the strong anti-oxidants biliverdin/bilirubin and the signaling molecule carbon monoxide. These versatile HO-1 products protect against ischemic and inflammatory injury. We postulate that orthodontic forces induce HO-1 expression in the PDL during experimental tooth movement. Twenty-five 6-week-old male Wistar rats were used in this study. The upper three molars at one side were moved mesially using a Nickel-Titanium coil spring, providing a continuous orthodontic force of 10 cN. The contralateral side served as control. After 6, 12, 72, 96, and 120 h groups of rats were killed. On parasagittal sections immunohistochemical staining was performed for analysis of HO-1 expression and quantification of osteoclasts. Orthodontic force induced a significant time-dependent HO-1 expression in mononuclear cells within the PDL at both the apposition- and resorption side. Shortly after placement of the orthodontic appliance HO-1 expression was highly induced in PDL cells but dropped to control levels within 72 h. Some osteoclasts were also HO-1 positive but this induction was shown to be independent of time- and mechanical stress. It is tempting to speculate that differential induction of tissue protecting- and osteoclast activating genes in the PDL determine the level of bone resorption and hyalinization and, subsequently, “fast” and “slow” tooth movers during orthodontic treatment.

## Introduction

Although orthodontic tooth movement has been described in multiple studies the exact biological mechanisms are still not elucidated. Large differences in the rate of orthodontic tooth movement are found between individuals in identical experimental settings and are largely independent of the magnitude of the orthodontic force (Pilon et al., 1996; Iwasaki et al., 2006; Van Leeuwen et al., 2010).

The PDL contains a variety of cells, blood vessels, nerves, and extracellular matrix (ECM) molecules. Forces applied to a tooth compress the blood vessels at the resorption side, while dilating them at the apposition side leading to inflammation and subsequent remodeling of periodontal tissues. At the resorption side, recruited osteoclasts will degrade the alveolar bone allowing tooth movement (Krishnan and Davidovitch, 2009; Sanuki et al., 2010; Kook et al., 2011), whereas at the apposition side signaling pathways are activated to stimulate precursor cells in the PDL to differentiate into osteoblasts (Park et al., 2010; Tamura et al., 2010).

Unfortunately, at the resorption side, ischemic injury can result in cell death in a process called hyalinization. At these hyalinized regions of the PDL no living osteoclasts are present to facilitate alveolar bone resorption. The necrotic tissue needs to be removed by macrophages and multinucleated giant cells, and to be replaced by newly formed connective tissue. However, these multinucleated giant cells and macrophages can also initiate root resorption, one of the most important challenges in orthodontics (Brudvik and Rygh, 1994; von Bohl and Kuijpers-Jagtman, 2009). Orthodontic patients can be classified as “fast” or “slow” tooth movers, indicating variance in response of the periodontal ligament (PDL) to hyalinization formation (van Leeuwen et al., 1999).

Protection against inflammatory, mechanical, and ischemic stresses, as present during hyalinization, may determine the efficacy of individual orthodontic tooth movement. Interestingly, it was recently observed that differential levels of the osteoclast activating cytokine IL-1β also modulate orthodontic tooth movement (Iwasaki et al., 2006; Cao et al., 2016). “Fast tooth movers” may have decreased levels of hyalinization via higher induction of cytoprotective genes and/or increased levels of osteoclast activating genes. Heme oxygenase-1 (HO-1) is one of the most important examples of cytoprotective genes against ischemia reperfusion injury (Katori et al., 2002; Wagener et al., 2003). Protection against ischemic injury and cell death by HO-1 overexpression is demonstrated for example in liver and heart transplantations (Exner et al., 2004). HO-1 degrades heme into free iron, carbon monoxide (CO) and biliverdin (Grochot-Przeczek et al., 2012). Biliverdin is then directly converted into bilirubin by biliverdin reductase. CO is a signaling molecule that regulates inflammation, angiogenesis, and apoptotic signaling. Biliverdin and bilirubin are both potent antioxidants, while free iron is scavenged by co-induced ferritin (Babusikova et al., 2008). The level of HO-1 induction following a stimulus varies within the human population because of its highly polymorphic promoter (Wagener et al., 2008).

HO-1 is induced by a wide array of stresses (Wagener et al., 2003). In an *in vitro* study, HO-1 induction by mechanical stress in PDL cells was previously demonstrated (Cho et al., 2010). In the present study we aim to translate this *in vitro* finding of HO-1 induction in the PDL cells to an *in vivo* experimental setting in rats using orthodontic forces. We postulate that HO-1 will be induced by orthodontic forces and subsequently reduces or prevents hyalinization formation and root resorption. Protection against those unwanted side effects may ameliorate orthodontic tooth movement.

## Materials and methods

### Experimental animals

Twenty-five 6-week-old male Wistar rats were used in this study. The animals were housed under normal laboratory conditions with *ad libitum* access to water and powdered rodent chow (Sniff, Soest, The Netherlands). The animals were allowed to acclimatize for at least 1 week before the start of the experiment. Ethical permission for the study was obtained according to the guidelines of the Board for Animal Experiments of the Radboud University Nijmegen (Reference number: RU-DEC-2006-160).

### Force application

The rats were at random divided into 5 groups, with 5 animals per group. A split-mouth design was used to control for individual variances. The three maxillary molars on one randomly chosen side were moved mesially as one unit by means of a coil spring as described previously (Ren et al., 2003; Xie et al., 2009). The contralateral maxillary molars served as controls. A Nickel-Titanium (Ni-Ti) 10 cN Santalloy® closing coil spring (GAC, New York, NY, USA) was attached to the molar block via incisor anchorage to deliver a constant mesially directed force. An important advantage of this biomechanical design is that a constant low force per molar was applied preventing tipping of the molars. The effect could be estimated as a force of 170 cN per human molar which is within the range used in the clinic. This design has been proved to be functional and caused tooth movement during an period of 12 weeks (Ren et al., 2004).

After 6, 12, 72, 96, and 120 h of force application, groups of rats were killed and perfused with 4% paraformaldehyde (PFA) in phosphate-buffered saline (PBS). The maxillae were removed, post-fixed for 24 h in 4% PFA, decalcified in 10% EDTA, and embedded in paraffin.

### Haematoxylin-eosin staining of maxillary parasagittal sections

Serial parasagittal sections (5 μm) mounted on Superfrost Plus slides (Menzel-Gläser, Braunschweig, Germany) were routinely stained with haematoxylin and eosin (HE) for general tissue survey. For immunohistochemical evaluation, sections containing at least the radicular pulp of three roots of the maxillary molars were selected.

### Immunohistochemical staining

Immunostaining for HO-1 and CD68 was done essentially as previously described (Tan et al., 2009). CD68 is a marker for cells of the macrophage lineage, including monocytes, giant cells and osteoclasts and is recognized by the ED1 antibody (Sminia and Dijkstra, 1986). Cells within the PDL were counted as osteoclasts when they were ED1-positive, multinuclear, and located at the alveolar bone outline.

In brief, tissue sections were deparaffinized and rehydrated, followed by quenching of endogenous peroxidase activity using 3% H_2_O_2_ in methanol. After post-fixation in 4% PFA, antigen retrieval was performed by heating sections in 10 mM citrate pH 6 at 70°C for 10 min, followed by incubation in 1% trypsin (Difco Laboratories, Detroit, MI) at 37°C for 7 min. Sections were subsequently blocked in 10% normal donkey serum (NDS; Chemicon, Temecula, CA) before overnight incubation at 4°C with primary antibody diluted in 2% NDS. The primary antibodies were polyclonal rabbit anti-HO-1 antibody (1:600; SPA-895, Stressgen, Ann Arbor, MI) and monoclonal mouse anti-CD68 ED1 antibody (1:200; MCA341R, Serotec, Breda, The Netherlands). Then sections were incubated with biotin-conjugated donkey anti-rabbit or anti-mouse secondary antibodies (1:3000–1:5000; Jackson Labs, West Grove, PA) for 1 h, followed by 45 min incubation with Vectastain ABC-Elite kit (Vector Laboratories, Burlingame, CA). Immunostaining was visualized by incubating the sections for 10 min with 3′3′-diaminobenzidine tetrahydrochloride (DAB; Sigma, St. Louis, MO). Staining was intensified with 0.5% CuSO_4_, and counterstained with haematoxylin. Photographs were taken on a Carl Zeiss Imager Z.1 system (Carl Zeiss Microimaging Gmbh, Jena, Germany) under bright field conditions.

### Analysis of immunohistochemical data

**Figure 1:**
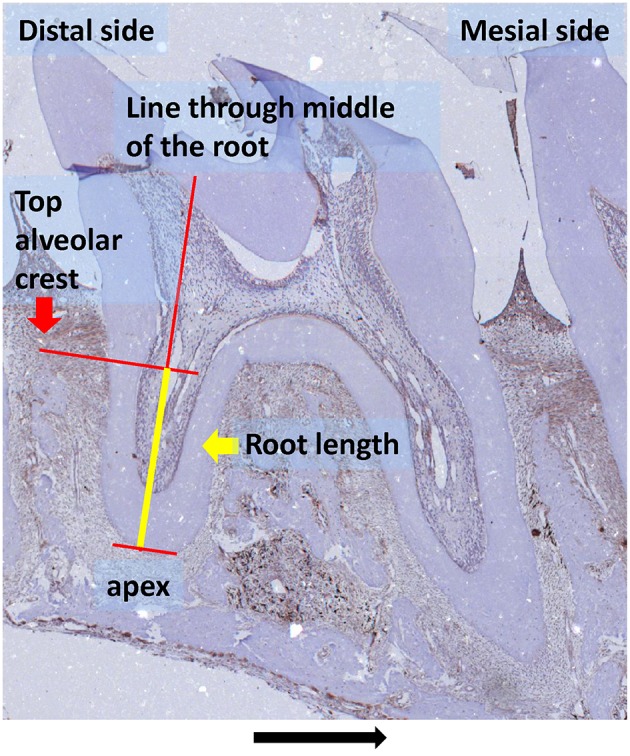
**Root length measurement (yellow line) as used for determining the number of cells/mm root explained in detail: root length was measured from apex to the top of the alveolar crest at the line through the middle of the root (HO-1 immunostaining, magnification: x25, experimental group 120 h, black arrow indicated the direction of the tooth movement)**.

The PDL region containing the roots of first and second molars was selected for quantitative measurement of HO-1 positive cells. Roots fulfilling the following criteria were included in the study: (1) The root structure is present from the cementoenamel junction to the apex, (2) the radicular pulp is present in the root structure, (3) no artifacts are present in the PDL structure. Almost all third molars were excluded because the root structure was not present over the whole length of the root in most sections. For standardization, root length was measured from apex to the top of the alveolar crest at the line through the middle of the root by using an ocular graticule (Figure 1). This allowed us to quantify the number of HO-1 and ED1 positive cells per mm of root length.

HO-1 positive mononuclear PDL cells and ED1 positive multinuclear osteoclasts were counted along the mesial and distal areas of the PDL in both experimental and control sides by blinded observers. Because the three maxillary molars were moved mesially as one unit, the mesial areas of all roots at the experimental sides were considered resorption sides, whereas the distal areas were considered apposition sides (Supplementary Figure 1). To determine the inter-examiner reliability, 10 sections were measured by the two observers and acceptable *R*^2^ > 0.80 were obtained for cell counting and root length measurements. For positively stained mononuclear and osteoclast cells, data was presented as the number of positive cells per mm of root length.

### Statistical analysis

The data from HO-1 positive mononuclear cells for control samples showed a normal distribution as measured by the Kolmogorov-Smirnov test (KS-test). No time dependency throughout the experimental period and no side dependency by one-way analysis of variance (ANOVA) was found. Therefore, these data were pooled and used as baseline HO-1 expression in PDL.

The data from both HO-1 positive mononuclear PDL cells for experimental samples and the control and experimental osteoclasts (HO-1 and ED1) showed a non-normal distribution and was analyzed using non-parametric Kruskal-Wallis ANOVA on ranks and Mann-Whitney tests.

The data for the HO-1 positive osteoclasts at the distal area of the control samples was normally distributed. Therefore, ANOVA and Tukey's multiple comparison *post-hoc* test were used to analyze the time dependency.

Independent-Samples *T*-test was performed to compare differences between osteoclasts for each marker (HO-1 and ED1) at the distal areas. Differences were assumed to be significant if *p* < 0.05.

## Results

### Effects of orthodontic force on HO-1 expression in PDL cells

Histological evaluation of HE-stained sections demonstrated that application of mechanical stress significantly reduced the width of the PDL at the resorption side, whereas the width at the apposition site was increased (Supplementary Figure 1). This confirms that the Ni-Ti coil spring appliance produced a mesially-orientated force acting on the PDL and the molar block. Hyalinized regions in the PDL were only observed at the resorption side in the experimental group (**Figure 5**), but not in the control group.

**Figure 2:**
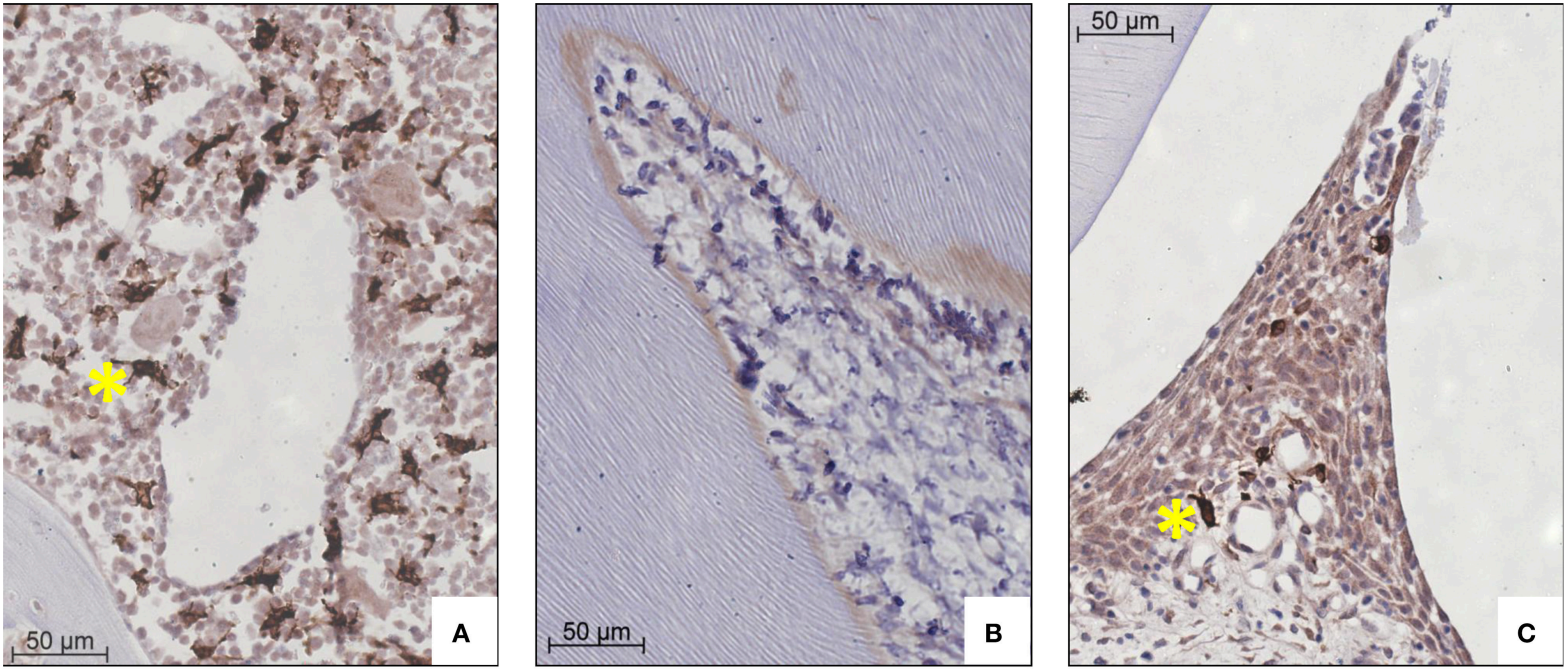
**HO-1 expression in bone marrow, pulp tissue and interdental papilla tissue representative for both control and experimental group (HO-1 immunostaining, magnification: x400, yellow asterisk indicates a HO-1 overexpressing cell). (A)** Large numbers of HO-1 overexpressing cells in bone marrow (control group 120 h). **(B)** Negligible numbers of HO-1 positive cells were present in pulp tissue (experimental group 12 h). **(C)** Few HO-1 positive cells in interdental papilla tissue (control group 120 h).

First we investigated the HO-1 expression in parasagittal sections at the control (no force) side. In these sections, HO-1 positive cells were strongly present in the bone marrow and only few HO-1 expressing cells were found in the PDL. The large number of cells expressing high levels of HO-1 in the bone marrow was used as an internal control for the staining procedure. Hardly any HO-1 positive cells were found in sections from pulp tissue, whereas interdental papillae were positive for HO-1 expressing cells (Figure 2).

**Figure 3:**
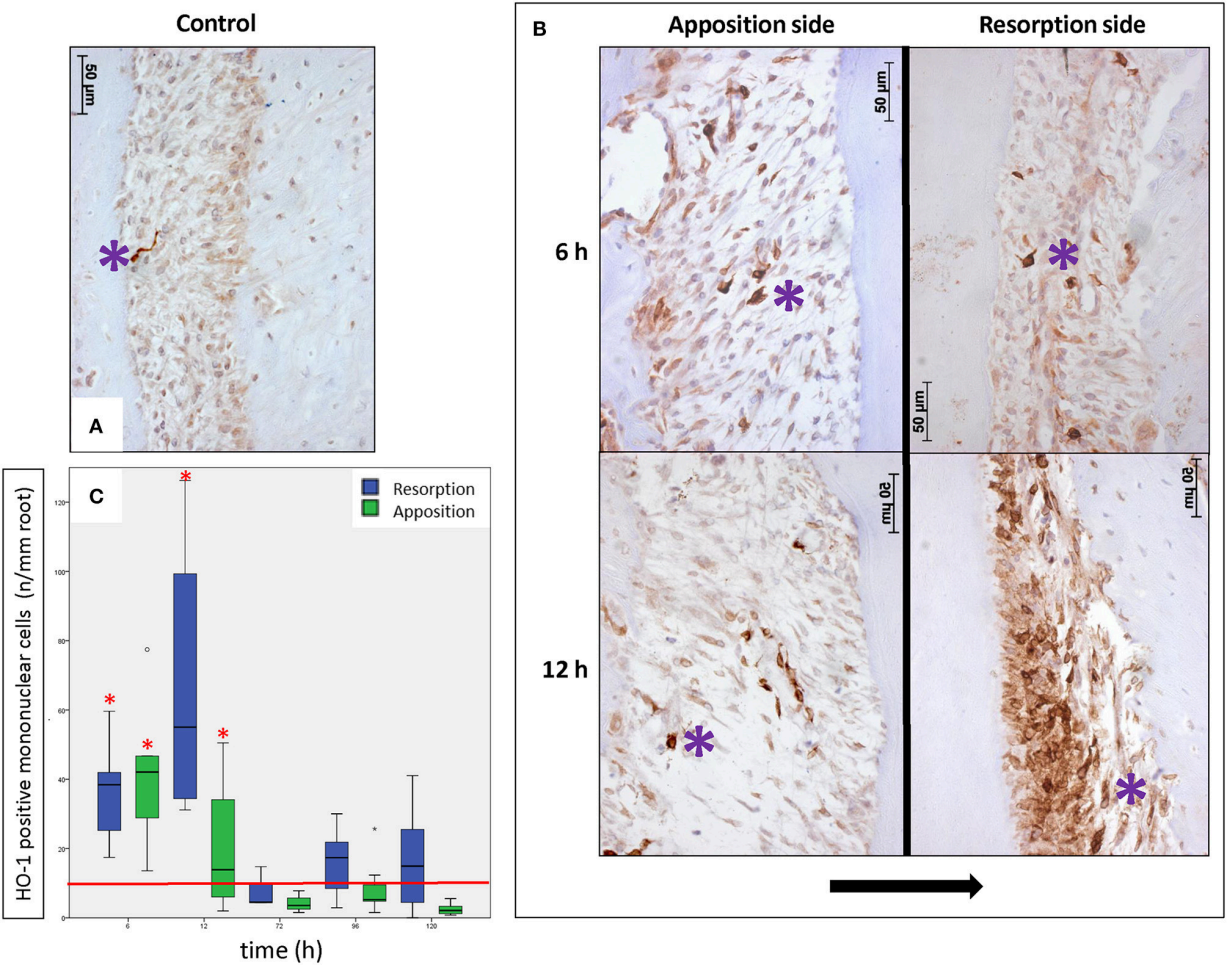
**Effects of orthodontic force on HO-1 expression in the PDL (HO-1 immunostaining, magnification: x400, purple asterisk indicates a HO-1 overexpressing cell, black arrow indicates the direction of the tooth movement). (A)** Only few strong HO-1 positive cells are observed in PDL tissue in control sections (control group 6 h). **(B)** Many HO-1 overexpressing cells were present in PDL tissue at both the apposition- and resorption side in experimental sections of experimental time 6 and 12 h. This was especially evident in the vicinity of blood vessels. **(C)** Box-plot of monunuclear PDL cells at the experimental sides compared to control. The number of HO-1 positive mononuclear PDL cells is significantly higher at 6 and 12 h of force application compared to the control group (base-line HO-1 expression in PDL, red horizontal line), for both the apposition- and resorption side (Kruskal-Wallis ANOVA on ranks tests, *p* < 0.001). ^*^Significantly different from base-line HO-1 expression in PDL, *p* < 0.05.

Next, we compared the effect of orthodontic force on HO-1 expression. We observed no change in the levels of HO-1 staining for the bone marrow, pulp and interdental papilla following mechanical stress at the measured time points. However, we detected a strong increase in the number of HO-1 positive mononuclear cells in the PDL after force application (Figure 3B) compared to the contralateral control side (Figure 3A). This was especially evident in the vicinity of blood vessels.

In order to quantify HO-1 protein in PDL tissue during orthodontic tooth movement experimental sections were examined and compared to controls. In general there were low numbers of cells strongly expressing HO-1 (< 10 cells/mm root) observed in the PDL at the control side. At the control side no statistically significant differences were detected between the number of HO-1 positive mononuclear PDL cells at the distal- and mesial side at any of the investigated time points nor between the different time points (*p* = 0.110). These data were therefore pooled, considered as base-line HO-1 expression in PDL (Figure 3C, red horizontal line).

At the experimental side there was a statistically significant time-dependent increase in the number of HO-1 positive cells per mm of root length and the data was therefore separated for the different time points and for both the resorption- and apposition side. Both for the resorption- and apposition side the number of HO-1 positively stained mononuclear PDL cells was significantly increased for the time points 6 and 12 h compared to no force application (*p* < 0.001, Figure 3C). Although the number of HO-1 positive stained mononuclear cells was higher at the resorption side after 12 h compared to the apposition side, this did not reach statistical significance (*p* = 0.083).

Summarizing, following orthodontic treatment, the PDL tissue showed a strong increase in the number of HO-1 positive cells at both the apposition- and resorption side compared to the contralateral control side, but returned within 72 h to baseline levels.

### HO-1 expression in osteoclasts

**Figure 4:**
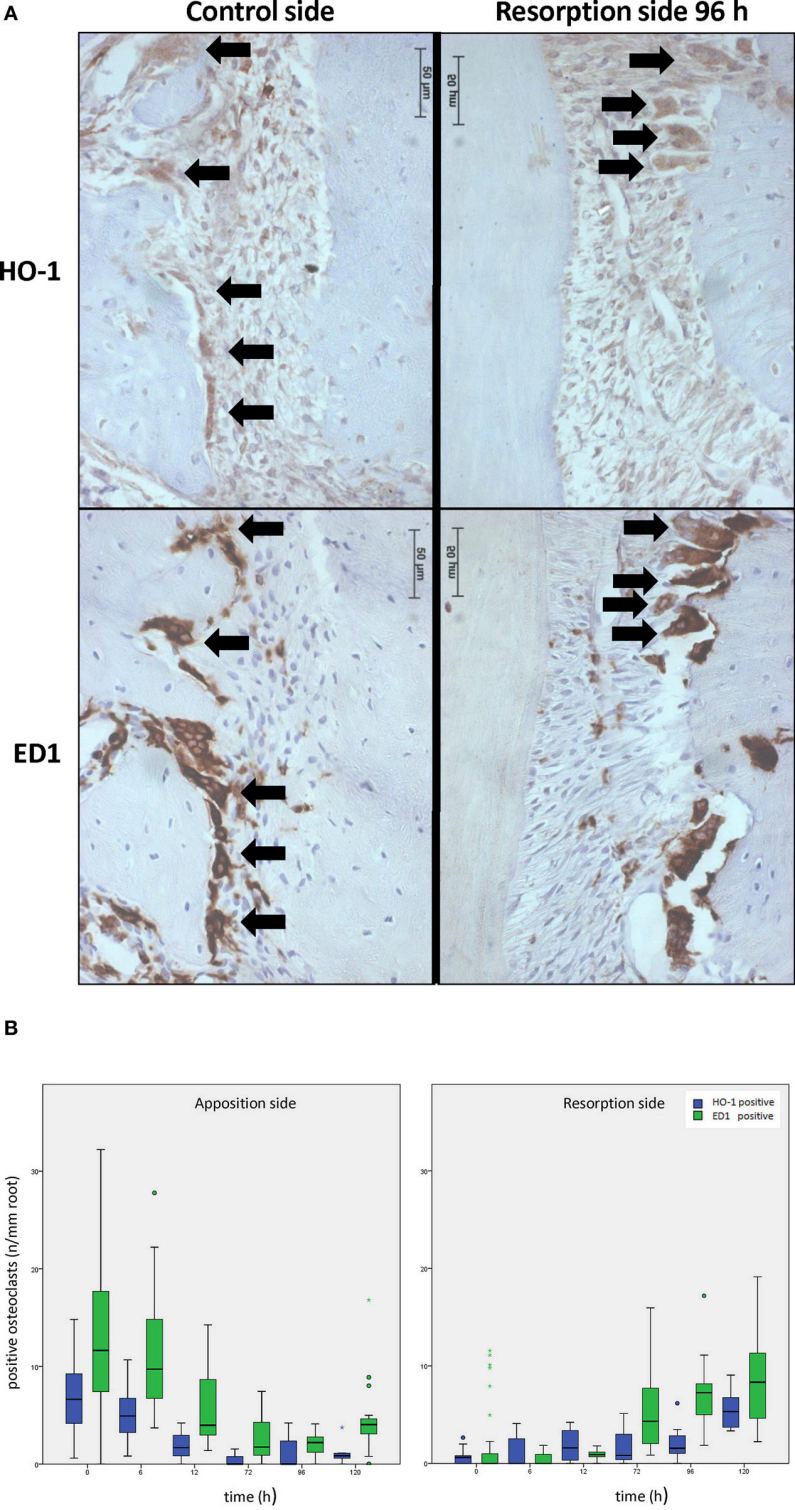
**Effects of orthodontic force on HO-1 and ED1 positive osteoclasts. (A)** HO-1 (upper lane) and ED1 (lower lane) positive osteoclasts are found both before [control group, distal side (left panel)] and after [experimental group 96 h, resorption side (right panel)] force application as shown by immunohistochemistry. (magnification: x400, black arrows indicating an HO-1 and ED1 positive osteoclast, cells that were multinuclear and located at the alveolar bone outline were marked as osteoclasts). **(B)** When no force is present osteoclasts are mainly present at the distal site. Upon mechanical stress, ED1 or HO-1 positive osteoclast numbers decrease at the distal side, what is now called the apposition side (left panel), whereas the amount of osteoclasts at the resorption side increase (right panel). HO-1 expression levels in the osteoclasts and the HO-1/ED1 ratio remain similar following mechanical stress (Independent-Samples *T*-tests, *p* > 0.05).

When no force was applied, osteoclasts were mainly observed in the bone marrow and in the PDL at the distal side (molar distal drift in rats, Supplementary Figure 1). However, upon mechanical stress, the number of osteoclasts within the PDL decreased at the distal side (apposition side), whereas at the resorption side the osteoclast numbers increased. A portion of the observed osteoclasts were also positively stained for HO-1 (Figure 4A). In control sections, HO-1 positively stained osteoclasts were observed in both the PDL and the bone marrow. The HO-1 expression levels in osteoclasts were, however, less intense compared to those in the HO-1 positive mononuclear PDL cells and bone marrow cells (Figures 2–4A).

In control sections the number of HO-1 and ED1 positive osteoclasts per mm of root length was significantly higher at the distal side compared to the mesial side (Figure 4B). The data were therefore separated for both areas.

When applying the mesially directed force, changes in the numbers of osteoclasts were observed at both sides (Figure 4B). After 120 h the numbers of HO-1 positive (*p* = 0.349) and ED1 positive (*p* = 0.065) osteoclasts found at the mesial side (resorption side) were similar to the distal side in control sections, and with a similar HO-1/ED1 ratio. In conclusion, expression of HO-1 in osteoclasts was demonstrated to be independent of time- and mechanical stress.

## Discussion

We demonstrated here for the first time that applying an orthodontic force strongly induces expression of the cytoprotective enzyme HO-1 in PDL cells at both the resorption- and apposition side in rats. Morphological changes within the PDL as a result of ischemic stress at the resorption side (compression of blood vessels, hyalinization areas) and mechanical stress at the apposition side (stretched blood vessels and increased PDL width) were observed (Supplemental Figure 1). In general the control side demonstrated relatively low numbers of HO-1 positive cells. Only bone marrow cells showed constitutively high expression levels of HO-1. Orthodontic force strongly induced HO-1 expression in cells situated closely around blood vessels, suggesting that these cells may be pericytes or infiltrated leukocytes (Zeng et al., 2015). A major part of the HO-1 positive PDL cells are fibroblasts, while only a minor part of osteoblasts and cementoblasts are HO-1 positive. In PDL cells HO-1 induction occurred shortly after placing the orthodontic appliance but dropped within 72 h to control values. Thus, although the orthodontic force was applied continuously HO-1 induction within the PDL cells was only temporary. Induction of HO-1 by mechanical stress was previously demonstrated in several *in vivo* (Rawlinson et al., 1998) and *in vitro* (Cho et al., 2010) experimental settings but not yet during orthodontic tooth movement.

Osteoclasts are bone-specific, indicating the existence of different subsets of osteoclasts at different bone-sites (Everts et al., 2009; Henriksen et al., 2011). Induction of HO-1 inhibited osteoclastogenesis *in vitro* and *in vivo* in arthritis models (Zwerina et al., 2005). It was demonstrated that induction of HO-1 in osteoclast precursors resulted in reduced osteoclast differentiation and subsequently decreased bone resorption. It was also shown that the majority of osteoclasts attached to bone erosions in human joints from patients with rheumatoid arthritis were negative for HO-1 (Zwerina et al., 2005). If these findings of osteoclasts in an arthritic setting could be seen in the oral setting as well, this could have major impact on our understanding of both tooth movement and root resorption. Effects of orthodontic force on HO-1 expression in osteoclasts was therefore studied in more detail.

As demonstrated previously, after application of a mesially-directed force with a Ni-Ti coil spring almost all osteoclasts had disappeared from the distal side within 12 h, while at the mesial side (new resorption side) the number of osteoclasts increased and after 120 h the numbers were similar to the distal side in the control group (Xie et al., 2009). There giant cells are also multinuclear and ED1 positive, we cannot exclude that giant cells entering the PDL and accidentally located at the alveolar bone outline were marked as osteoclasts. However, we remain confident that the large majority of the observed multinuclear, ED1 positive alveolar bone lining cells were osteoclasts.

Both experimental- and control sides demonstrated comparable proportions of HO-1 positive osteoclasts. The proportions of HO-1/ED1 positive cells had not changed significantly, indicating that the basal levels of HO-1 expression in osteoclasts was independent of time and mechanical strain. Thus, mechanical stress did not induce HO-1 expression in osteoclasts. This was also true for the dental pulp cells, interdental papilla tissue, and bone marrow cells. Since large numbers of HO-1 positive osteoclasts are present in the bone marrow, where normal osteoclast differentiation occurs it is unlikely that osteoclastogenesis is hampered following force induction. In rheumatoid arthritis bone resorption is mainly a pathological process (Wagener et al., 2008), whereas alveolar bone resorption is a normal physiological adaptive process. Functional differences were indeed described between osteoclasts located at long flat bones (craniofacial region) and long bones (axial skeleton) with respect to their acid- and protease secretion during bone degradation (Everts et al., 2009; Henriksen et al., 2011).

Reduction or prevention of hyalinized tissue formation is thought to facilitate orthodontic tooth movement and to decrease undermining resorption. It is tempting to speculate that the levels of cytoprotective genes that are activated during orthodontic tooth movement determine whether tooth movement or hyalinization take place. HO-1 has been demonstrated to attenuate inflammation and to prevent tissue injury and apoptosis following ischemic injury (Zarjou et al., 2011; Kumar et al., 2013). Expression of this cytoprotective HO-1 within the PDL likely generates a protecting environment and subsequently inhibits ischemic injury (von Bohl and Kuijpers-Jagtman, 2009).

**Figure 5:**
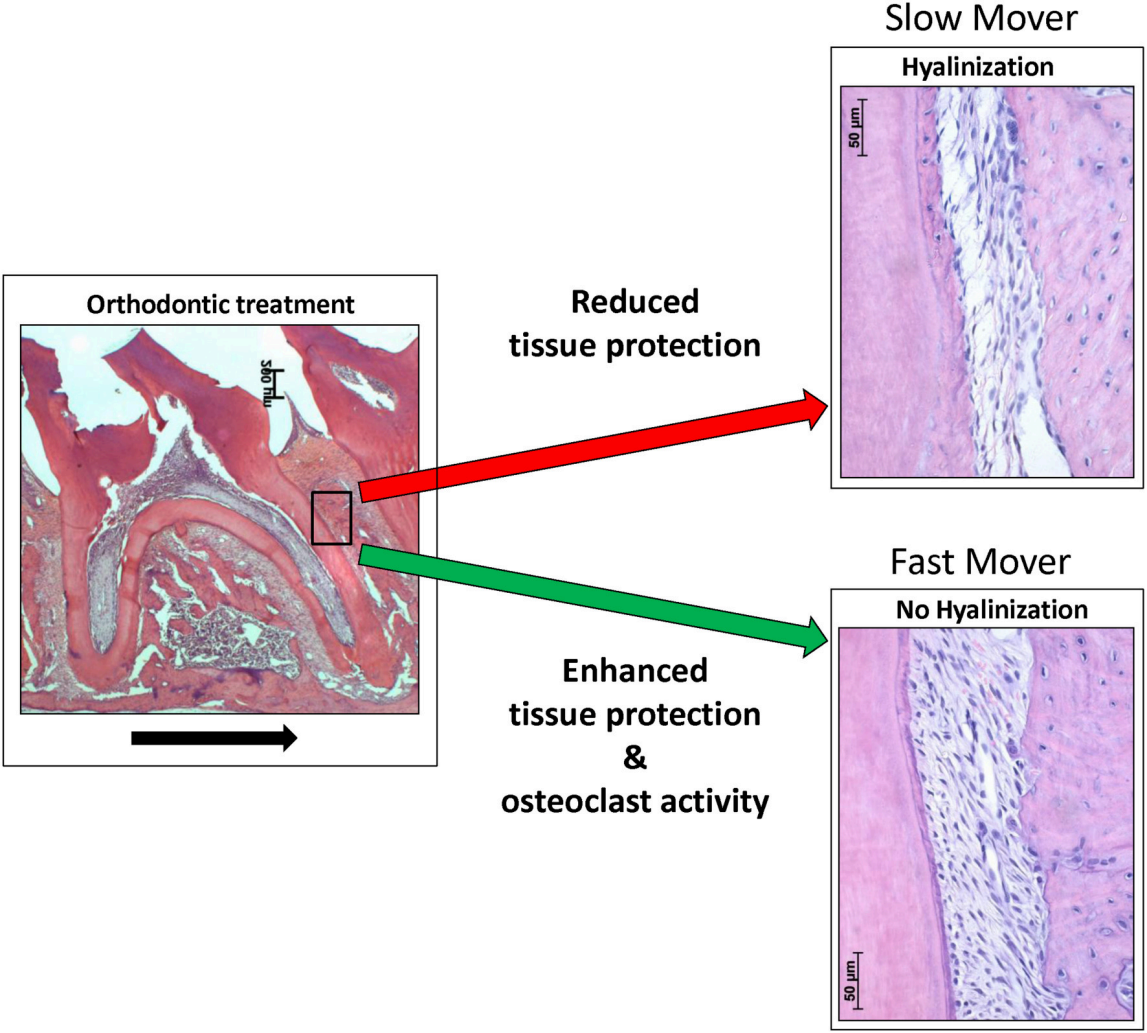
**Orthodontic force can result in hyalinization- or no hyalinization formation in the PDL, resulting in “slow” or “fast” tooth movers**. In this hypothetical model we postulate that differential expression of tissue protective genes determine, in concert with osteoclast activating genes, the tooth movement. Applied orthodontic force results in mechanical stress within the PDL. This mechanical stress induces expression of HO-1 in PDL cells. Reduced HO-1 expression levels may lead to increased levels of ischemic injury and cell death (hyalinization), resulting in a hampered orthodontic tooth movement rate. Expression of cytoprotective enzymes, as HO-1, likely reduces ischemic injury and cell death. We hypothesize that differential expression of osteoclast activating genes (e.g., IL-1 β) and tissue protecting genes (e.g., HO-1) determines the remodeling of the PDL, or alveolar bone and therefore the orthodontic tooth movement rate. Differences in gene expression levels (e.g., as a result of polymorphisms) levels possibly explain the “slow” and “fast” tooth movers following orthodontic treatment. “Fast” tooth movers have enhanced osteoclastic activity (e.g., high IL-1 β) and protection against hyalinization (e.g., high HO-1), whereas “slow” tooth movers show hyalinization hampering osteoclast activity (black arrow indicates the direction of the tooth movement).

In humans the levels of HO-1 induction are largely controlled by a promoter polymorphism, that has been shown to have strong clinically significance (Exner et al., 2004; Wagener et al., 2008). The existence of “slow” and “fast” tooth movers (van Leeuwen et al., 1999) could thus possibly be explained by differences in individual expression of HO-1. The HO-1 promoter polymorphism in humans, determining the levels of HO-1 induction, are not present at the equivalent positions in the rat and mouse ho-1 genes (Shibahara et al., 2002). The influence of gene polymorphism of IL-1β at the tooth movement rate has been demonstrated previously in orthodontic patients (Iwasaki et al., 2006). Therefore, we hypothesize that remodeling of the PDL and alveolar bone is determined by the sum of the expression of osteoclast activating- and tissue protecting genes in the PDL microenvironment. Differences found in tooth movement rate could be explained by differential expression of these genes (Figure 5). Interestingly, HO-1 induction is attenuated at higher age (Abraham and Kappas, 2008), which may further explain the increased levels of root resorption and hyalinization after orthodontic treatment at older age.

Since HO-1 was only shortly induced during the application of the continuous orthodontic force, this suggests that accommodation to the mechanical and ischemic stress takes place. Interestingly, when orthodontic forces are interrupted with inactive periods strongly reduced root resorption has been observed in rats and dogs (Kameyama et al., 2003). The mechanism of this protection remains unknown. Possibly, renewal of HO-1 induction occurs during intermittent force application, contributing to decreased root resorption and hyalinization formation. Further research to tooth displacement and hyalinization by modulating the HO-1 system is warranted to reveal the exact role of HO-1 during orthodontic tooth movement.

## Conclusion

Summarizing, the present study showed that orthodontic forces induced HO-1 expression in the PDL in rats in the mononuclear cell population. Although the force was applied continuously, the HO-1 expression was only temporarily. HO-1 positive osteoclasts are present but this expression was shown to be independent of time- and mechanical stress. HO-1 induction may be a novel target for reducing unwanted side effects of orthodontic force application in the future.

## Author contributions

CS: analyzed data, wrote manuscript. RX: performed experiments, edited manuscript. DL: analyzed data, wrote manuscript. AK: analyzed data, wrote manuscript. JU: performed experiments. RV: performed experiments, edited manuscript. JM: analyzed data, designed experiments, wrote manuscript. FW: analyzed data, designed experiments, wrote manuscript.

### Conflict of interest statement

The authors declare that the research was conducted in the absence of any commercial or financial relationships that could be construed as a potential conflict of interest.

## Supplementary material

Supplementary Figure 1 **Applied orthodontic force results in changes in morphology of the PDL. (A)** No orthodontic force application (HE staining, control molar, picture magnification: x25; distal- and mesial side, picture magnification: x400, control group 6 h). At the distal side irregular alveolar bone outline is present due to resorption by osteoclasts (molar distal drift in rats). At the mesial side a more regular alveolar bone outline is observed. **(B)** After 6 h of orthodontic force application (HE staining, orthodontic treatment 6 h picture magnification: x25, apposition- and resorption side picture magnification: x400) changes in the width of the PDL at both the mesial- and distal side were observed (black arrow indicates the direction of the tooth movement). At the mesial side the width of the PDL was reduced and bloodvessels of the PDL were compressed (resorption side). At the distal side the width of the PDL was increased and bloodvessels of the PDL were stretched (apposition side).
