# Supplementary figures and images for: Orthodontic Forces Induce the Cytoprotective Enzyme Heme Oxygenase-1 in Rats
Source: Front Physiol. 2016 Jul 19;7:283. doi: 10.3389/fphys.2016.00283 (PMC4949267; doi:10.3389/fphys.2016.00283)

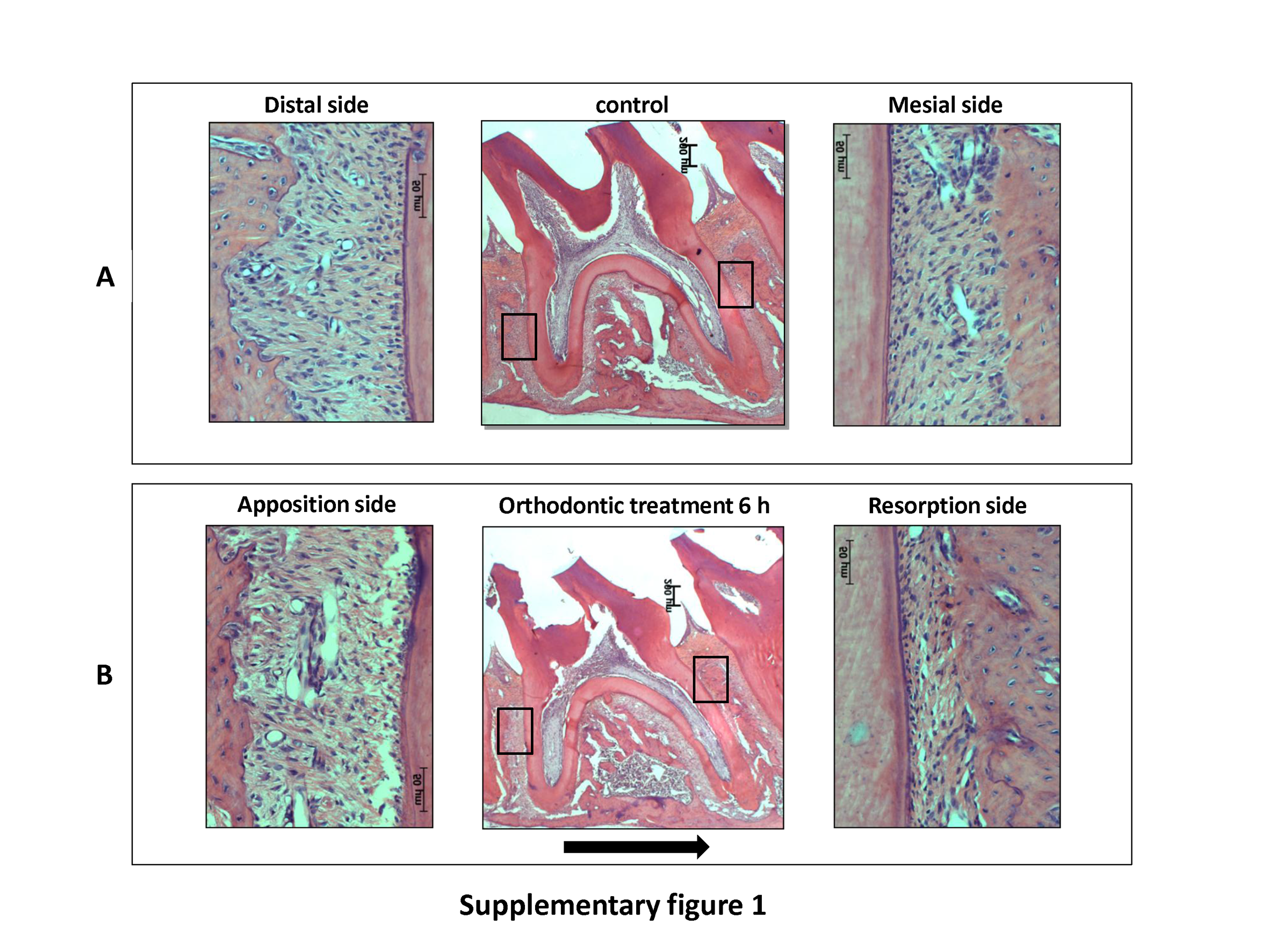

Supplement: Supplementary Figure 1 — Applied orthodontic force results in changes in morphology of the PDL. (A) No orthodontic force application (HE staining, control molar, picture magnification: x25; distal- and mesial side, picture magnification: x400, control group 6 h). At the distal side irregular alveolar bone outline is present due to resorption by osteoclasts (molar distal drift in rats). At the mesial side a more regular alveolar bone outline is observed. (B) After 6 h of orthodontic force application (HE staining, orthodontic treatment 6 h picture magnification: x25, apposition- and resorption side picture magnification: x400) changes in the width of the PDL at both the mesial- and distal side were observed (black arrow indicates the direction of the tooth movement). At the mesial side the width of the PDL was reduced and bloodvessels of the PDL were compressed (resorption side). At the distal side the width of the PDL was increased and bloodvessels of the PDL were stretched (apposition side). [file Image1.tiff]
